# Case Report: Multimodal contact immunotherapy in alopecia areata: long-term clinical experience in the JAK inhibitor era

**DOI:** 10.3389/fmed.2026.1757777

**Published:** 2026-02-24

**Authors:** Maira E. Herz-Ruelas, Miranda M. Contreras-Hernández, Ilse M. Salazar-Herz

**Affiliations:** 1 Christus Muguerza Hospital Sur, Monterrey, Mexico; 2 Universidad de Monterrey (UdeM), School of Medicine, San Pedro Garza García, Mexico; 3 Instituto Tecnológico y de Estudios Superiores de Monterrey (ITESM), School of Medicine, Monterrey, Mexico

**Keywords:** adjunctive therapy, alopecia areata, combined, immunotherapy, JAK inhibitor, multimodal

## Abstract

**Introduction:**

Alopecia areata (AA) poses a significant therapeutic challenge. While biologics and Janus kinase (JAK) inhibitors offer novel treatment options, immunotherapy remains a relevant approach. This case series describes the long-term clinical experience and the practical use of contact immunotherapy in managing alopecia areata in combination with other current treatment modalities.

**Methods:**

We present five patients with chronic alopecia areata who have been successfully treated with topical immunotherapy for 9–15 years in combination with other treatments, achieving excellent disease control and demonstrating long-term safety.

**Results:**

All patients achieved sustained long-term disease control with a multimodal approach.

**Conclusion:**

This case series highlights the enduring value of topical immunotherapy, even in the era of novel targeted therapies. It underscores the importance of individualized, multimodal approaches to optimize patient outcomes and demonstrates that traditional immunotherapy continues to be a valuable treatment option for alopecia areata.

## 1 Introduction

Alopecia areata (AA) is a non-scarring autoimmune disorder affecting the hair follicle. It has a global prevalence of 2%, with considerable clinical variation and severity. Although the majority of cases present as patchy AA, a smaller proportion progress to more extensive, chronic, and resistant subtypes (1). Despite remarkable therapeutic advances over the past decade, AA remains a challenging condition due to its unpredictable course, high recurrence rates, and significant psychological burden on those affected.

The emergence of Janus kinase (JAK) inhibitors has positively transformed the therapeutic landscape of AA, offering unprecedented treatment results in moderate and severe cases. Despite their high response rates, access to these agents remains limited due to their high cost, lack of insurance approval, restricted availability, and potential adverse effects. Consequently, traditional long-standing therapies, such as immunotherapy with contact allergens, continue to play an important role in current management strategies for AA.

Topical immunotherapy has been used since the 1970s in patients with refractory or extensive AA, yielding positive results (2). This approach is based on the principle of inducing allergic contact dermatitis, acting through immunomodulation via antigenic competition, and modulating lymphocytic activity against the hair bulb. Dinitrochlorobenzene (DNCB) was the first agent utilized, but it was later discontinued due to its mutagenic properties. Currently, either diphenylcyclopropenone (DPCP) or squaric acid dibutyl ester (SADBE) is used, with a response rate of 50–60%. The application of a contact sensitizer induces a type IV hypersensitivity reaction, redirecting the autoimmune response away from the hair follicle by promoting a shift from a Th1/IFN-*γ*-dominant response to a Th2/IL-10 profile. Skin treated with topical immunotherapy exhibits a decrease in the peribulbar CD4/CD8 lymphocyte ratio, reduction in the number of intra-bulbar CD6 + lymphocytes and Langerhans cells, and loss of the expression of class I and II major histocompatibility complex molecules that are normally present in AA patches (3, 4).

Immunotherapy for the treatment of AA has many advantages, such as being more affordable than newer, more expensive therapies, having no systemic side effects, requiring no monitoring studies, and possessing a favorable safety profile that allows for long-term management of this chronic condition.

Historically, immunotherapy for managing AA was primarily used as monotherapy. This was largely due to limited understanding of the underlying pathophysiology of the disease at the time and the lack of comprehensive therapeutic options in contemporary clinical practice. In contrast, the current range of trichologic therapies makes a combined approach highly appealing for optimizing clinical outcomes.

In light of the high cost of current therapies and their limited accessibility for some patients, this study describes our long-term, real-world experience with multimodal contact immunotherapy for AA. Extensive follow-up of five representative cases demonstrates the sustained clinical utility and safety of this therapeutic modality in combination with other accessible therapies, such as topical and oral minoxidil, intralesional and oral corticosteroids, antioxidants, and phototherapy, highlighting its enduring role in the era of JAK inhibitors.

## 2 Methods

Five patients with chronic or extensive AA treated with DPCP and/or SADBE in combination with adjunctive therapies were ambispectively analyzed. The follow-up period ranged from 9 to 15 years. Clinical responses, adjuvant therapies, and treatment outcomes are described.

After signing the informed consent form, the patients were sensitized with 2% DPCP or SADBE applied to a 5 cm^2^ area of the scalp. After 8 h, the immune sensitizer was removed, and the patient returned to the clinic 3 weeks later for weekly applications of gradually increasing concentrations (0.001% until 2%) of the topical sensitizer. Concentrations were adjusted according to the degree of elicited erythema, pruritus, and hair regrowth (5). When the maximum degree of regrowth was achieved, treatments were spaced every 2–3, or even 4, months, with application of the contact allergen to the entire scalp rather than being limited to alopecic areas.

A multimodal, individualized approach targeting complementary pathways in the treatment of AA and related hair disorders was employed, combining intralesional triamcinolone acetonide, oral dexamethasone, topical minoxidil at concentrations of 5–10%, high-dose oral minoxidil, supplements such as vitamin D, zinc, and quercetin. Additionally, phototherapy and topical or oral finasteride were included, with the goal of achieving more durable and cosmetically satisfactory results.

## 3 Results

We describe five illustrative clinical cases, four female and one male cases, aged 15 to 43 years at baseline, with long-term follow-up ranging from 9 to 15 years, all receiving multimodal contact immunotherapy (Table 1).

**Table 1:** Long-term outcomes in patients with alopecia areata treated using a multimodal approach.

Patient number	1	2	3	4	5
Gender	Female	Female	Female	Female	Male
Age at baseline	43 years old	37 years old	15 years old	27 years old	16 years old
Baseline type of alopecia areata/initial SALT score	AU/S100	AU/S100	AT/S85	AU/S100	Patchy alopecia areata/S65
Associated diseases/comorbidities	Vitiligo Autoimmune thyroid disease	None	Vitiligo	None	Vitiligo Autoimmune thyroid disease
Associated hair disorders	Female androgenetic alopecia	Female androgenetic alopecia	None	None	Male androgenetic alopecia
Disease duration to date	25 years	26 years	20 years	20 years	10 years
Multimodal combined approach: cumulative therapies to date	Oral minoxidil 5 mg Topical minoxidil 5% Intralesional triamcinolone Vitamin D DPCP, SADBE	Oral minoxidil 5 mg Topical minoxidil 5% Intralesional triamcinolone Vitamin D Zinc Oral dexamethasone pulses DPCP, SADBE	Oral minoxidil 5 mg Topical minoxidil 5% Intralesional triamcinolone Vitamin D Quercetin DPCP, SADBE	Oral minoxidil 5 mg Topical minoxidil 5% Intralesional triamcinolone Vitamin D Quercetin Excimer lamp SADBE	Oral minoxidil 5 mg Topical combined minoxidil 10%/finasteride 0.5 mg Intralesional Triamcinolone Vitamin D Excimer lamp LLLT Oral finasteride 1 mg DPCP
Response	Excellent cosmetic results, occasional relapses, currently isolated alopecic patches	Excellent cosmetic results, occasional relapses, persistent ophiasis pattern	Excellent cosmetic results, occasional relapses, persistent ophiasis pattern	Excellent cosmetic results, occasional relapses, currently isolated alopecic patches	Excellent cosmetic results, occasional relapses
Long-term outcome (maximum SALT score achieved-current-)	S0	S1	S1	S1	S0
Total follow-up	15 years	12 years	12 years	9 years	10 years

Patient characteristics. AU, alopecia areata universalis; AT, alopecia areata totalis; SADBE, squaric acid dibutyl ester; DPCP, diphenylcyclopropenone.

During the initial evaluation, three patients were diagnosed with alopecia universalis (AU). One patient, with a SALT score of 85, was classified as having alopecia totalis (AT) due to diffuse scalp hair loss characterized by small, scattered hair tufts and a few areas of higher density, with no involvement of the eyebrows, eyelashes, or body hair. The fifth patient presented with extensive patchy AA, with a SALT score of 65. Three patients had vitiligo, two of whom also had autoimmune thyroid disease. In addition, three patients had concomitant androgenetic alopecia, although only the male patient requested treatment for this condition as part of his therapeutic regimen.

**Figure 1:**
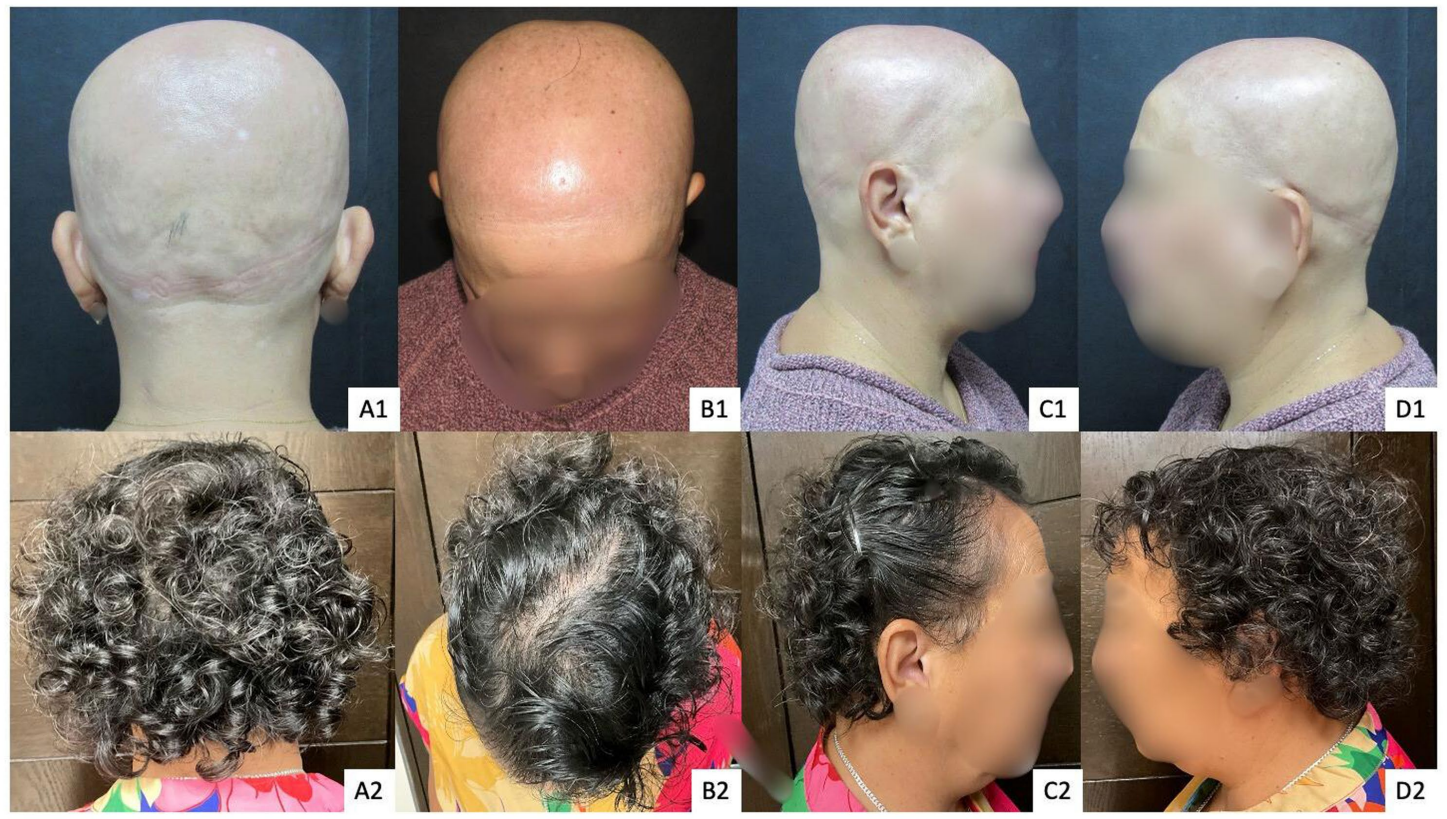
Patient 1 with AU. Panels **(A1–D1)** show baseline findings, and panels **(A2–D2)** show outcomes after 15 years of follow-up using immunotherapy as part of a combined multimodal approach.

**Figure 2:**
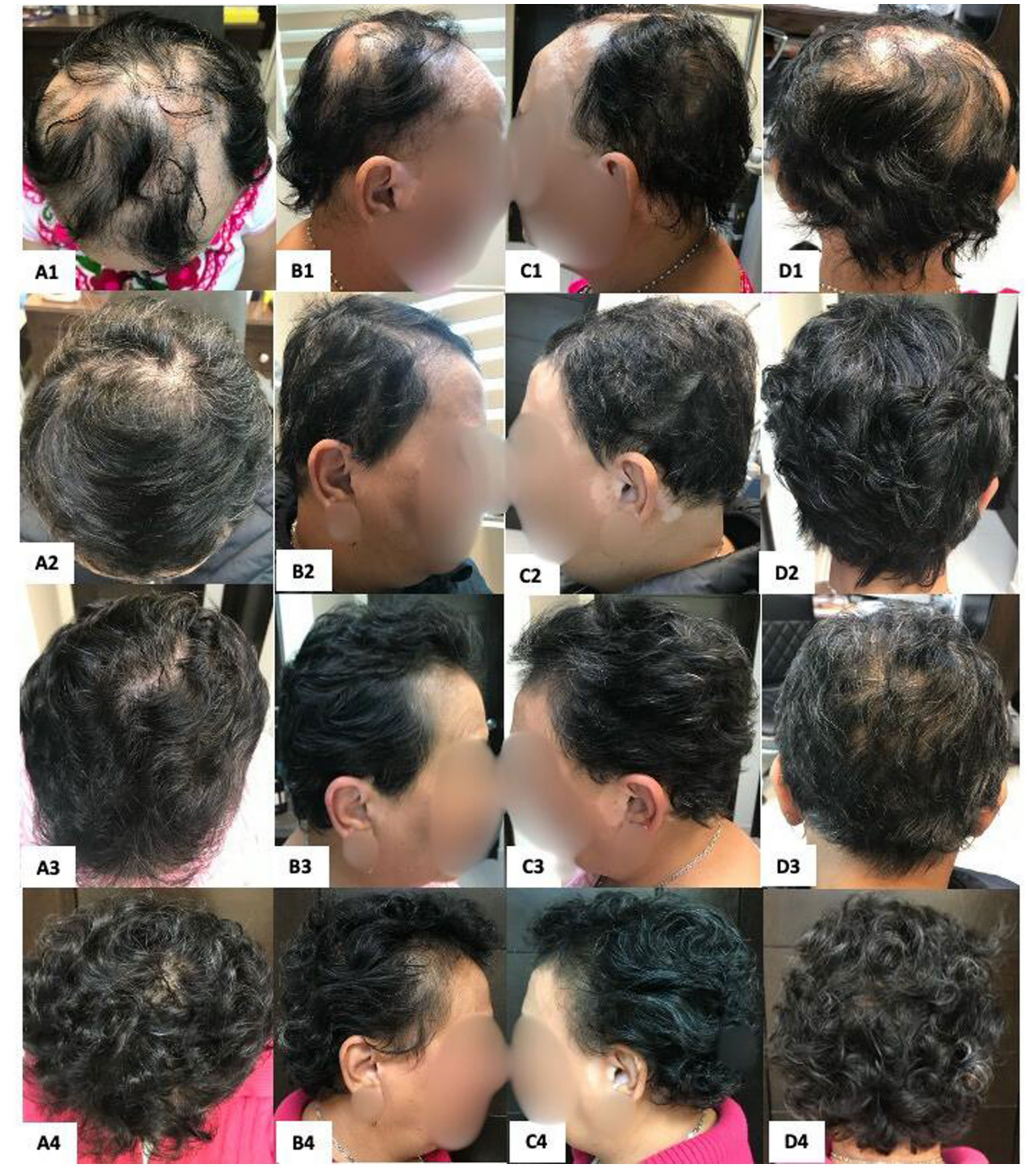
Patient 1 with AU. Panels **(A1, B1, C1, D1)** show the baseline relapse. Panels **(A2, B2, C2, D2)** show the patient after 4 months of SADBE 2% plus 2.5 mg/mL intralesional triamcinolone every 6 weeks (after three treatments). Panels **(A3, B3, C3, D3)** show outcomes after 2 months of SADBE 2%. Panels **(A4, B4, C4, D4)** show outcomes after 4 months of SADBE 2%. The patient is currently on maintenance therapy with SADBE 2% applied to the entire scalp every 3–4 months.

The multimodal approach involved the simultaneous, continuous, or sequential use of therapies, including triamcinolone acetonide injections at selected sites during relapse for faster recovery or in persistent lesions (Figures 1, 2), oral dexamethasone pulses as a rescue therapy during severe relapse, topical minoxidil at concentrations of 5–10%, high-dose oral minoxidil up to 5 mg, supplements such as vitamin D, zinc, and quercetin, and excimer lamp therapy. Low-level laser therapy (LLLT), along with topical and oral finasteride, was utilized in the male patient who wished to address androgenetic alopecia as part of his therapeutic regimen.

**Figure 3:**
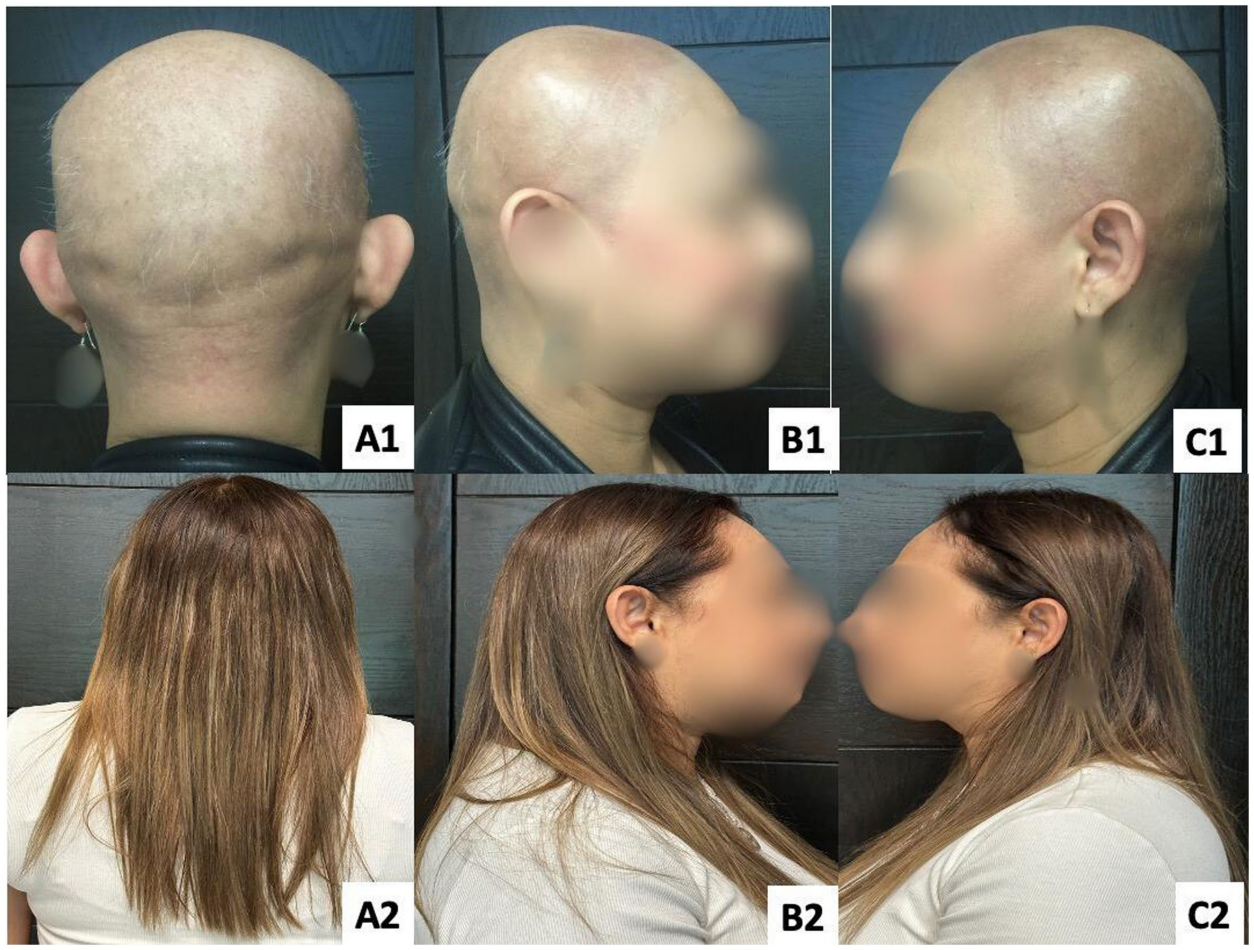
Patient 2 with AU. Panels **(A1–C1)** show baseline findings, and panels **(A2–C2)** show outcomes after 12 years of follow-up using immunotherapy as part of a combined multimodal approach.

**Figure 4:**
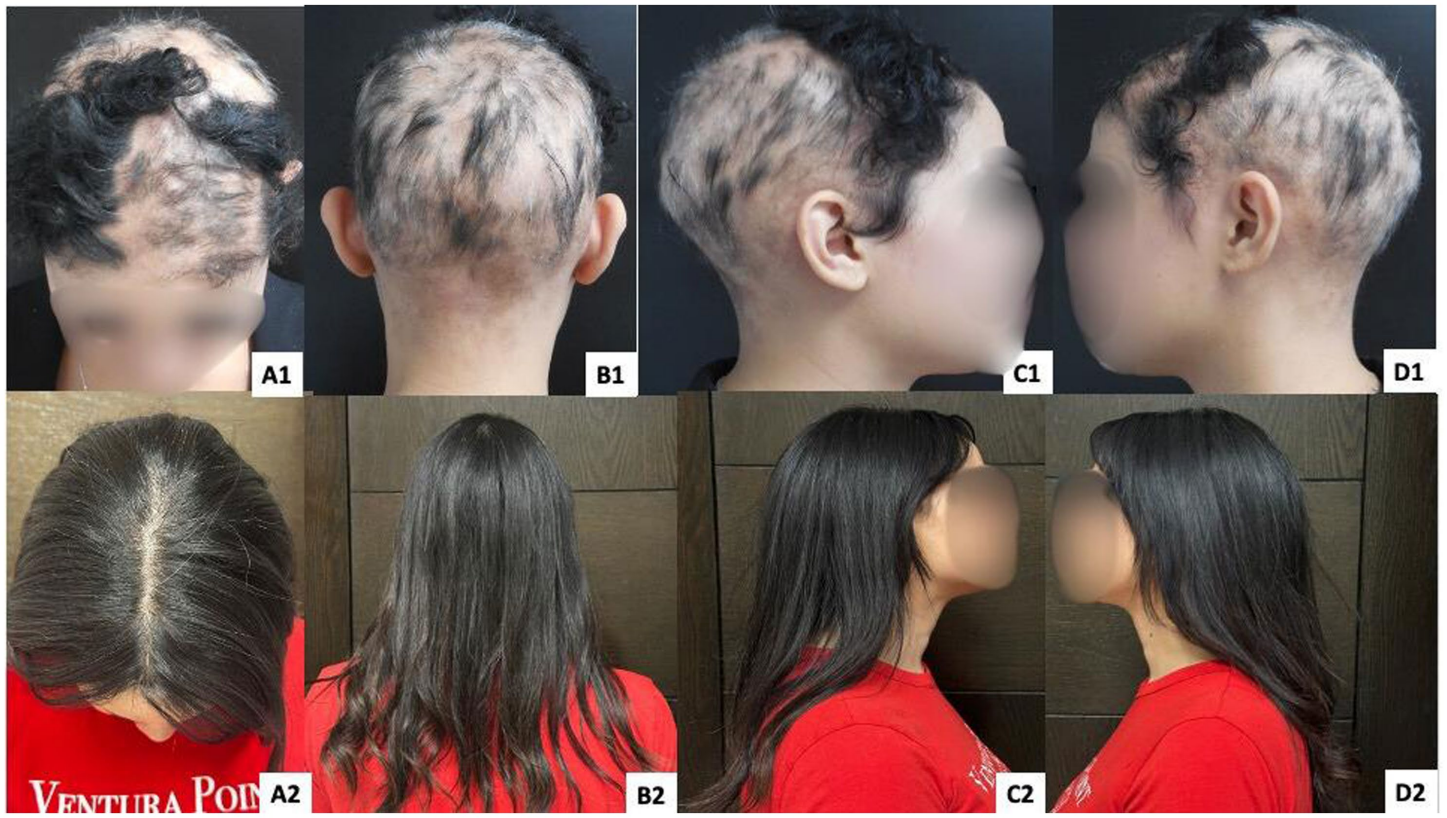
Patient 3 with AT. Panels **(A1, B1, C1, D1)** show baseline findings, and panels **(A2, B2, C2, and D2)** show outcomes after 12 years of follow-up using immunotherapy as part of a combined multimodal approach.

All patients achieved adequate disease control. Two patients currently have a SALT score of 0, while three have a SALT score of 1. The latter group exhibited excellent cosmetic outcomes, as the few residual, scattered alopecic patches were effectively concealed by mature, fully regrown hair, resulting in optimal camouflage. Only minor adverse effects were observed, which were limited to contact dermatitis associated with SADBE or DPCP immunotherapy. Three patients initially received immunotherapy with DPCP; however, due to inadequate response, they were transitioned to sensitization with SADBE, resulting in successful hair regrowth (Figures 1, 3, and 4). The author previously reported a 50% probability of response when switching to an alternative sensitizing agent following initial treatment failure (5).

No laboratory monitoring was required, except for patient 2, who received oral dexamethasone pulses at a dosage of 0.1 mg/kg/day, administered 2 days per week. Dexamethasone was gradually tapered over a 6-month period, based on the patient’s clinical progress. Systemic dexamethasone was utilized as rescue therapy for a severe relapse, and this intervention was required only once throughout the treatment and follow-up period (Figure 3).

All patients continued their ongoing treatment with systemic minoxidil at a dosage of 5 mg per day. They started treatment with a lower dose when fine vellus hairs began to grow. This strategy was implemented to promote a prolonged anagen phase, thereby enhancing their hair length and thickness. In each case, the dosage of minoxidil was gradually increased as tolerated, up to a maximum of 5 mg. On average, the patients required 1–3 rescue treatments per year with 2.5 mg intralesional triamcinolone acetonide injections for localized large and/or persistent AA patches.

The frequency of topical immunotherapy applications was adjusted and shortened according to each patient’s treatment response. In cases of relapse, the frequency of immunotherapy applications was even increased to weekly sessions, followed by spacing out the applications as needed based on ongoing clinical assessments.

Long-term safety, efficacy, and adaptability of multimodal contact immunotherapy were demonstrated in all five patients with AA.

## 4 Discussion

Although JAK inhibitors represent a breakthrough, their high cost, limited accessibility, and potential side effects restrict the widespread use. Therefore, despite being a ¨classical¨ therapy, contact immunotherapy offers a safe, cost-effective, topical alternative that can be combined with currently available therapies, such as topical and oral minoxidil, oral and intralesional corticosteroids, excimer light therapy, and hair supplements.

This synergistic combination leverages complementary mechanisms, including immune modulation, anti-inflammatory effects, and enhanced follicular regeneration. This multifaceted approach is thus posited to not only mitigate relapse frequency but also promote more durable and aesthetically superior outcomes.

Minoxidil, initially developed as an antihypertensive, is a well-established therapy for AA. Its primary action involves opening ATP-dependent potassium channels, leading to vasodilation and increased cutaneous blood flow. This enhances the delivery of oxygen and growth factors to the hair follicle and stimulates prostaglandin E2 (PGE2) and leukotriene B4 (LTB4) production. These effects collectively promote continuous hair follicle growth, extend the anagen phase, and shorten telogen shedding. In addition to its vascular effects, minoxidil also exhibits immunomodulatory properties that likely contribute to its efficacy in treating AA. It modulates concanavalin A-induced T-cell suppression and acts as a potent proliferative and anti-apoptotic agent on human dermal papilla cells. This action is mediated through the activation of ERK and Akt pathways, the elevation of the Bcl-2/Bax ratio to prevent cell death, and the downregulation of IL-1α gene expression.

A systematic review and meta-analysis evaluating the efficacy of monotherapy with minoxidil in AA included 372 patients. After analyzing 13 eligible articles, the review found a response rate of 82% for 5% topical minoxidil (95% CI 0.7–0.93) and 58% (95% CI 0.5–0.67) for concentrations below 5%. For the group of patients treated orally, the response rate was also 82% (6).

Systemic corticosteroids administered as pulse therapy have been used to treat AT/AU, with success rates ranging from 36 to 75% (7). Pulse therapy is effective and has a lower incidence of adverse effects. Treatment protocols using oral prednisolone, intravenous methylprednisolone, oral betamethasone, intramuscular triamcinolone, and oral dexamethasone have been reported (7–11). A dosage of 0.1 mg/kg/day administered biweekly has been reported as a potential therapeutic option, achieving a complete response in 71% and a partial response in 10% of 31 AU/AT patients (12).

Each milligram of dexamethasone is equivalent to 6.7 mg of prednisone, with a relative glucocorticoid potency of 25–30 and a relative mineralocorticoid potency of 0. The duration of action is long, lasting between 36 and 54 h, which allows its use in conjunction with topical immunotherapy, typically 2 days before the application of DPCP or SADBE. The desired effect of these contact sensitizers is expected to occur within 48 h, ensuring no interference with the mechanism of action of the immunotherapy (13).

Intralesional corticosteroids are still considered the first-line treatment for localized AA. A systematic review and meta-analysis of 69 eligible articles, including 543 participants with focal AA, evaluated intralesional steroid concentrations ranging from 2.5 to 10 mg/mL, administered every 3 to 4 weeks for periods of 6 weeks to 6 months. For intralesional triamcinolone acetonide, pooled hair regrowth rates were 62.3% for less than 5 mg/mL, 80.9% for 5 mg/mL, and 76.4% for 10 mg/mL (14).

Intralesional corticosteroids exert an anti-inflammatory effect on the characteristic peribulbar lymphocytic infiltrate in AA, thereby potentially mitigating the desired contact dermatitis induced by the topical sensitizer. In patients with stable immunotherapy who experience a flare, focal intralesional corticosteroid injections can be administered directly to affected areas while continuing immunotherapy across the entire scalp surface. This approach preserves the efficacy of the sensitizing agent in areas not receiving intralesional steroids. Furthermore, whether topical sensitizers exert their effect locally or systemically is still unknown. In most treated individuals, hair regrowth occurs at the application site, but a distant effect can also be observed, including regrowth of eyebrows, eyelashes, facial hair, or body hair (3). This phenomenon supports the potential for combined approaches in relapse cases where focal intralesional steroid administration may be appropriate.

Excimer light therapy can be delivered via an excimer laser or an excimer lamp, both emitting light using a wavelength of 308 nm in the ultraviolet range. Nuclear DNA absorbs ultraviolet B radiation, and p53 is upregulated, destroying DNA and inducing cell apoptosis, which inhibits dermal proliferation and reduces perifollicular inflammation and damage. Advantages include targeted treatment and relatively few adverse reactions (15). A systematic review concluded that 50.2% of patients achieved cosmetically acceptable hair regrowth after an average of 12 weeks (16).

As with other immune-mediated disorders, AA may result from a complex interplay between environmental factors and genetic predisposition. Deficiencies in micronutrients such as vitamins and minerals could represent modifiable risk factors linked to the development of AA. Therefore, supplementation of these nutrients as an adjunctive treatment may offer potential benefit. Notably, levels of vitamin D and zinc are often lower in patients with AA compared to healthy controls (17). Evidence indicates that vitamin D acts as a pivotal immunomodulator in AA, playing a crucial role in regulating follicular immune privilege and essential inflammatory pathways. Its deficiency has been correlated with increased disease severity, longer duration, and a higher risk of relapse. Emerging research supports the therapeutic potential of vitamin D supplementation in AA as a promising adjunctive therapy (18).

Zinc is important for DNA stability and repair mechanisms that are essential for maintaining normal hair growth. Its deficiency has been investigated as a contributing factor in autoimmune diseases and may play a role in AA pathogenesis. Statistically lower zinc levels have been identified in patients with AA, correlating with disease severity. Therefore, zinc supplementation could play a role in the restoration of hair follicles (19).

The effects of quercetin, a bioflavonoid with anti-inflammatory properties, were tested on the development of AA in the C3H/HeJ mouse model and the expression of HSP70, a heat shock protein involved in inflammatory response. Mice with AA were treated with subcutaneous quercetin. Hair regrowth was observed in all quercetin-treated mice. In addition, non-alopecic C3H/HeJ mice were subjected to heat-induced alopecia, along with quercetin injections. None of the mice receiving quercetin injections developed alopecia, and the level of HSP70 expression in quercetin-treated areas was comparable to that of the control. Intraperitoneal injections prevented/reduced the spontaneous onset of AA. Quercetin may serve as an effective adjuvant for the treatment and prevention of recurrent AA (20).

A multifaceted approach addressing associated hair conditions in patients with AA—including immunotherapy, correction of identified vitamin and mineral deficiencies, incorporation of antioxidants, and concurrent use of anti-inflammatory and immunomodulatory therapies with complementary mechanisms of action—may enhance therapeutic outcomes, potentially leading to improved aesthetic results, better disease control, and a reduction in the risk of relapse.

Although the long-term follow-up reported in this case series supports the enduring applicability of immunotherapy for AA, especially when used as part of a multimodal approach, the findings should not be interpreted as evidence for generalizable conclusions. Rather, they represent a descriptive account of five patients with an exceptionally long follow-up period, during which a favorable clinical course was observed. This manuscript aims to describe real-world clinical experience in the management of challenging cases, particularly in situations where JAK inhibitors are not an option due to contraindications, limited availability, or restricted access—circumstances that are especially relevant in resource-limited or developing healthcare settings.

We acknowledge that the small sample size precludes the extraction of objective, generalizable conclusions, as standardized outcome measures were not systematically collected as required for a formal clinical study. Accordingly, this report does not aim to propose a standardized therapeutic strategy or protocol, but rather to document the long-term clinical management of these patients in routine clinical practice over many years.

Furthermore, no comparative assessment of efficacy with contemporary therapies—many of which are more practical and demonstrably effective—was intended or performed. We therefore emphasize the descriptive nature of this manuscript, recognizing its inherent limitations, including the small sample size, the absence of systematically collected objective outcomes, and the lack of a comparator group.

We conclude that contact immunotherapy with DPCP or SADBE remains a valuable tool in the long-term management of this chronic and unpredictable disease. Its inclusion in multimodal regimens remains important, especially in clinical settings where biologic therapies and JAK inhibitors are unavailable, unaffordable, or contraindicated.

## Data Availability

The original contributions presented in the study are included in the article/supplementary material, further inquiries can be directed to the corresponding author.
